# A Hybrid YOLO and Segment Anything Model Pipeline for Multi-Damage Segmentation in UAV Inspection Imagery

**DOI:** 10.3390/s25216568

**Published:** 2025-10-25

**Authors:** Rafael Cabral, Ricardo Santos, José A. F. O. Correia, Diogo Ribeiro

**Affiliations:** 1 CONSTRUCT—iRail, Faculty of Engineering, University of Porto, 4200-465 Porto, Portugal; 2 iBuilt, School of Engineering, Polytechnic of Porto, 4249-015 Porto, Portugal

**Keywords:** structural inspection, damage segmentation, segment anything model, YOLO, deep learning, activation map

## Abstract

The automated inspection of civil infrastructure with Unmanned Aerial Vehicles (UAVs) is hampered by the challenge of accurately segmenting multi-damage in high-resolution imagery. While foundational models like the Segment Anything Model (SAM) offer data-efficient segmentation, their effectiveness is constrained by prompting strategies, especially for geometrically complex defects. This paper presents a comprehensive comparative analysis of deep learning strategies to identify an optimal deep learning pipeline for segmenting cracks, efflorescences, and exposed rebars. It systematically evaluates three distinct end-to-end segmentation frameworks: the native output of a YOLO11 model; the Segment Anything Model (SAM), prompted by bounding boxes; and SAM, guided by a point-prompting mechanism derived from the detector’s probability map. Based on these findings, a final, optimized hybrid pipeline is proposed: for linear cracks, the native segmentation output of the SAHI-trained YOLO model is used, while for efflorescence and exposed rebar, the model’s bounding boxes are used to prompt SAM for a refined segmentation. This class-specific strategy yielded a final mean Average Precision (mAP50) of 0.593, with class-specific Intersection over Union (IoU) scores of 0.495 (cracks), 0.331 (efflorescence), and 0.205 (exposed rebar). The results establish that the future of automated inspection lies in intelligent frameworks that leverage the respective strengths of specialized detectors and powerful foundation models in a context-aware manner.

## 1. Introduction

The escalating deterioration of global civil infrastructure, particularly concrete structures, presents a critical challenge to public safety and economic stability. National assessments consistently highlight the urgent need for maintenance and repair, with a significant portion of assets rated in poor condition [1]. The structural integrity of this infrastructure is often compromised by surface-level damage such as cracks, efflorescences, and exposed rebars. Traditionally, the identification and evaluation of this damage have relied on manual visual inspections. However, this long-standing practice is inherently problematic; it is not only time-consuming and costly but is also prone to significant inconsistencies and errors stemming from the subjective judgment of human inspectors [2]. To overcome these limitations, the field of Structural Health Monitoring (SHM) has explored various advanced techniques, ranging from model-based stiffness identification [3] to the vision-based approaches that are the focus of this work.

This paradigm is being reshaped by the integration of Unmanned Aerial Vehicles (UAVs) and advanced computer vision [4,5]. UAVs have revolutionized data acquisition for structural health monitoring, enabling the safe, rapid, and large-scale collection of high-resolution imagery, even in difficult-to-access locations [6,7]. The availability of high-fidelity visual data has catalysed the development of deep learning models for automated analysis. Initial efforts successfully applied Convolutional Neural Networks (CNNs) for image-level classification, effectively determining the presence of damage like cracks [8]. The field rapidly progressed to object detection models, such as the You Only Look Once (YOLO) family [9] and Faster R-CNN [10], which not only classify but also localize damages within bounding boxes, advancing beyond simple classification. These models have proven effective for identifying a wide range of defects on concrete [11], steel [12], and even specialized components like railway fasteners [13,14]. However, for accurate engineering analysis, quantifying the severity of damage, including its precise length, area, and shape, is essential. This requirement necessitates a more granular, pixel-level understanding of the image content.

**Figure 1:**
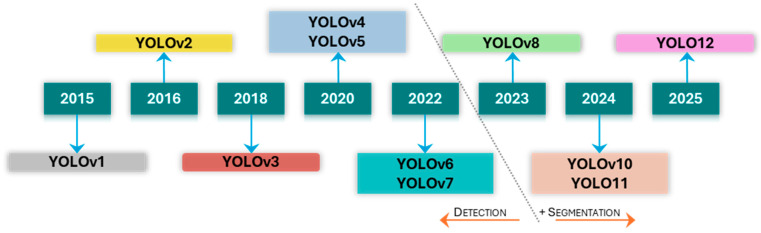
Evolution of the YOLO series.

This need for detailed geometric characterisation has driven the adoption of semantic and instance segmentation techniques. Architectures such as Fully Convolutional Networks (FCN) [15], U-Net [16], and DeepLabv3+ [17] established the foundation for pixel-wise classification. More recent models, including Mask R-CNN [18] and the segmentation-enabled versions of the YOLO series starting from YOLOv8, as illustrated in Figure 1, have further advanced this capability [19]. While these supervised models demonstrate high performance, their efficacy is fundamentally constrained by two critical bottlenecks. The first is the need for vast quantities of meticulously annotated data; the process of manually creating pixel-perfect masks for training is exceptionally laborious and requires domain expertise. To mitigate this bottleneck, some research has focused on synthetic data generation to create large, automatically annotated datasets that augment limited real-world data [20,21,22]. The second is the domain shift created by high-resolution UAV imagery; models trained on standard, lower-resolution public datasets often fail to generalize to the unique scale and textural detail present in aerial data [23].

The work by Lemos, Cabral [6] provides a compelling case study of confronting these issues. They developed a methodology combining UAVs and the Mask R-CNN framework to automatically segment corrosion on industrial roofing systems. To address the data bottleneck, they created a large, dedicated database of over 8000 high-resolution images with more than 18,000 annotated instances. More importantly, their work directly addressed the image processing challenge. They found that simply resizing high-resolution images to fit model input dimensions was detrimental, causing small corrosion instances to become “imperceptible to the algorithm, resembling only noise”. This led to a significant drop in performance, with the resized-image model failing to detect small anomalies that the high-resolution cropped-image model successfully identified. By adopting a cropping strategy that preserved resolution and carefully optimizing their architecture, they ultimately achieved a mean Average Precision (mAP50) of 59.2% for mask segmentation, highlighting that both dataset scale and appropriate high-resolution processing are critical for effective damage assessment.

The recent introduction of foundation models, particularly the Segment Anything Model (SAM), offers a promising solution to the data-scarcity problem [24]. Pre-trained on a dataset of over one billion masks, SAM exhibits remarkable zero-shot segmentation capabilities, generating precise masks from simple user prompts like points or bounding boxes without task-specific training. This has opened new frontiers for data-efficient analysis. Initial applications in civil engineering have explored a synergistic approach, coupling an object detector (e.g., YOLO) with SAM, where the detector’s bounding box is used as a spatial prompt to guide the final segmentation [6,25,26].

Building on this, newer approaches have explored a two-stage process that leverages the distinct strengths of different foundation models. Santos and Carvalho [26] demonstrated this by first employing a YOLOv8 object detection model to identify multiple classes of structural damage on bridges, such as cracks, rust stains, and efflorescence and then using the resulting bounding boxes to guide the powerful Segment Anything Model (SAM) for precise instance segmentation with mAP50 of 0.951. In a similar manner, Rakshitha, Srinath [25] successfully integrated the Mask R-CNN framework to generate bounding box prompts for SAM, which significantly improved the accuracy of crack segmentation on pavement datasets, achieving a mean Intersection over Union (mIoU) score of up to 0.83. However, it is important to note that direct comparison of these studies remains challenging, as they address different structural contexts and lack a common benchmark dataset for standardized evaluation.

While this two-stage approach is a logical first step, it suffers from a fundamental limitation when applied to the high-resolution imagery characteristic of modern inspections. For geometrically complex and elongated defects like cracks, a bounding box is an inefficient and often ambiguous prompt. A tight bounding box around a thin crack contains a large percentage of background pixels, providing a noisy signal to SAM that can result in incomplete or inaccurate segmentation. This represents a need for a more intelligent and context-aware prompting mechanism that can fully leverage the detail in high-resolution images and transcend the limitations of simple bounding boxes.

This study presents a comprehensive comparative analysis designed to identify the optimal deep learning strategy for segmenting structural damage in high-resolution UAV imagery. The analysis is twofold: first, it quantitatively benchmarks the performance of varying architectures within the YOLO11 series to establish the most effective foundational detector for the target domain. Second, leveraging the optimized YOLO11 model, it evaluates the efficacy of a novel two-stage framework by systematically comparing three distinct instance segmentation strategies: the model’s native segmentation output, the SAM prompted with conventional bounding boxes, and SAM prompted with a refined approach using precise point prompts derived from the detector’s internal probability map. The objective is to determine which approach is most robust and accurate, thereby establishing a clear benchmark for the application of foundation models in automated structural inspection.

## 2. Methodology

### 2.1. Framework Overview

**Figure 2:**
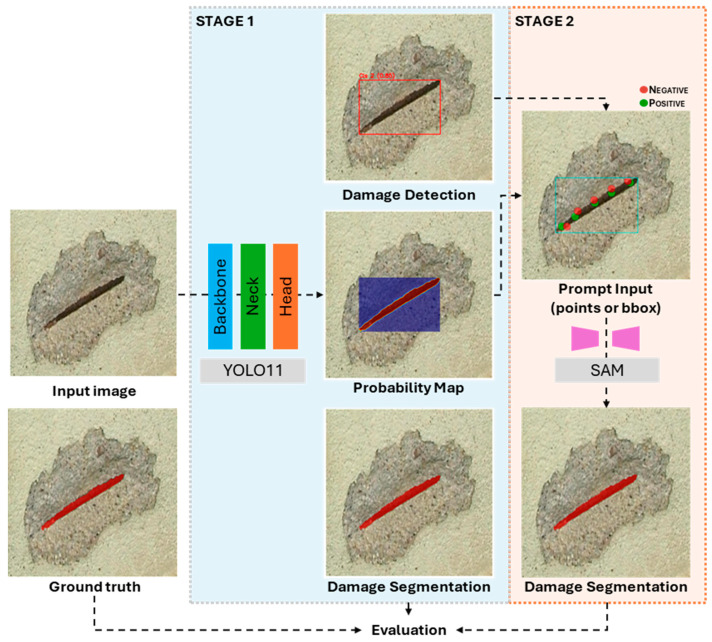
An overview of the proposed two-stage segmentation framework.

The proposed methodology is structured as a sequential, two-stage framework designed to achieve high-precision damage segmentation by synergizing a deep learning object detector with a foundational segmentation model. This approach aims to evaluate segmentation accuracy for complex damage patterns while minimizing the dependence on extensive pixel-level manual annotation. The framework employs a fine-tuned You Only Look Once (YOLO) model for initial damage identification and the Segment Anything Model (SAM) for the final segmentation. The overall process is depicted in Figure 2.

The initial stage of the framework involves processing an input image with a fine-tuned YOLO model. The purpose of this stage is to perform rapid and accurate multi-class damage identification, localising damage such as cracks, exposed rebar, and efflorescence within bounding boxes. The key innovation of this framework lies in the subsequent step: instead of utilizing the bounding box coordinates as a direct prompt for the segmentation model, a class-specific activation map is generated from the YOLO model’s internal representations. This map provides a spatial heatmap that highlights the image regions most influential to the detector’s classification decision for each specific damage instance.

In the second stage, the framework leverages the activation map to generate a segmentation mask using SAM. An optimised point-prompting strategy is applied to the activation map, which involves identifying the highest-confidence pixel. These calculated pixels serve as precise and contextually relevant spatial prompts that guide the SAM. By leveraging these focused cues, the model produces a detailed pixel-level segmentation mask that accurately delineates the true boundaries of the detected damage, effectively isolating it from the surrounding background.

### 2.2. Stage 1: YOLO-Based Damage Detection and Activation Map Generation

#### 2.2.1. YOLO Model Architecture

**Figure 3:**
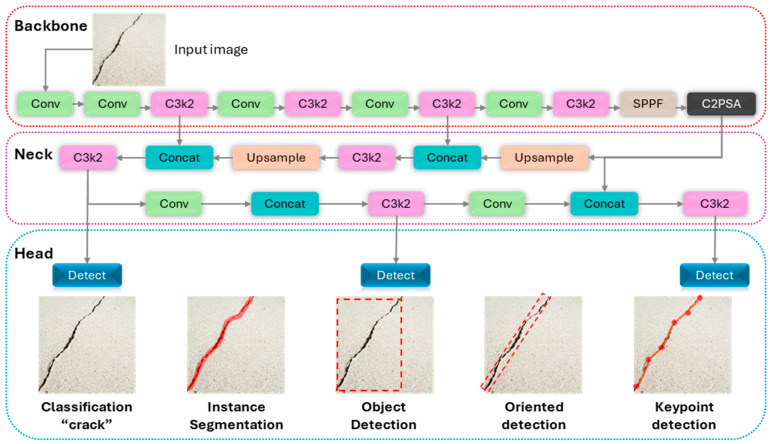
YOLO11 model architecture.

The You Only Look Once version 11 (YOLO11) represents a significant evolution in the YOLO series, building upon the highly successful and versatile framework of YOLOv8. The architecture is engineered to enhance the balance between detection accuracy and computational efficiency, making it suitable for a wide range of real-world applications. It retains the proven modular structure of its predecessors, which is conceptually divided into three principal components: the Backbone, the Neck, and the Head. The key innovations in YOLO11 are concentrated in the introduction of more efficient feature extraction blocks and the integration of an advanced self-attention mechanism. The YOLO11 model architecture is depicted in Figure 3.

**Figure 4:**
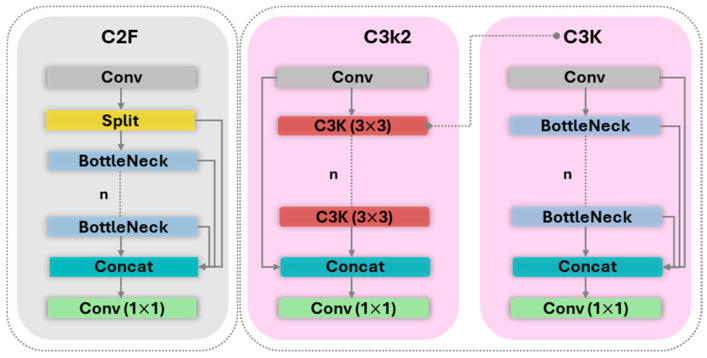
Difference of C2F and C3K2 Blocks.

**Backbone.** The Backbone of a detection network serves as the primary feature extractor, processing the input image through a series of convolutional layers to generate a rich hierarchy of feature maps. In YOLO11, the principal architectural enhancement is the replacement of YOLOv8’s C2f block with the novel C3k2 block. As depicted in Figure 4, the C3k2 block is a more parameter-efficient evolution of the Cross-Stage Partial (CSP) concept that refines information flows through the network. This optimization improves feature representation and enhances model efficiency without compromising its deep feature extraction capabilities.

**Neck.** The Neck is designed to aggregate and fuse the feature maps produced by the backbone at different scales. This multi-scale feature fusion is critical for enabling the model to accurately detect objects of various sizes and resolutions within a single image. The YOLO11 neck leverages two powerful components: Spatial Pyramid Pooling Fast (SPPF) and Cross-Stage Partial with Self-Attention (C2PSA).

**Figure 5:**
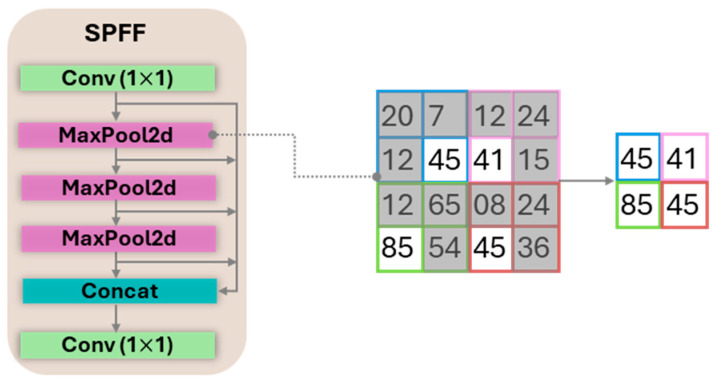
SPPF Block with a representative max-pooling operation.

To achieve a multi-scale receptive field with minimal computational cost, YOLO11 uses SPPF module positioned at the end of the backbone. As detailed in Figure 5, the module processes a feature map through several parallel max-pooling layers with different kernel sizes and concatenates the outputs. This process allows the model to capture rich contextual information at various scales efficiently.

**Figure 6:**
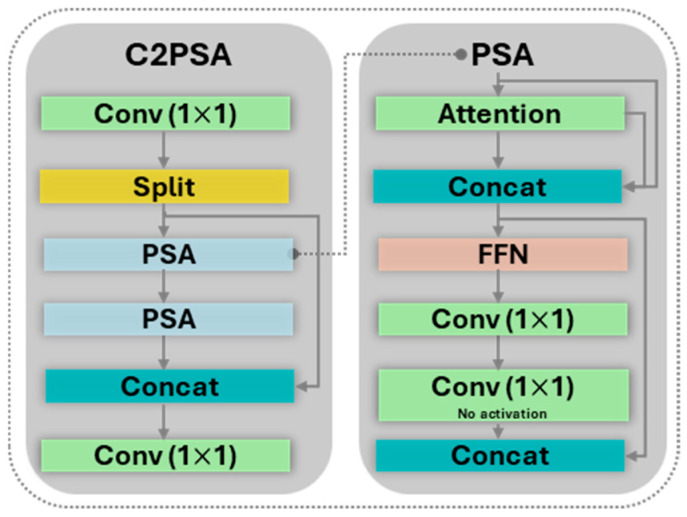
C2PSA block.

The YOLO11 neck integrates an advanced self-attention mechanism via the Cross-Stage Partial with Self-Attention (C2PSA) module. The structure of this block, shown in Figure 6, allows the network to dynamically weigh the importance of different spatial regions in the feature maps, enabling it to focus on the most informative pixels while suppressing irrelevant background noise. To optimize the trade-off between performance and computational cost, the C2PSA block is strategically placed after the SPPF module, where the feature map’s spatial resolution is lowest.

**Head.** The Head of the YOLO11 is an advanced, multi-task framework that generates the final predictions. A key evolution from previous versions, its modular design supports a variety of computer vision tasks beyond standard anchor-free Object Detection. As illustrated in the final stage of Figure 3, the head can be configured with additional branches for Instance Segmentation, Oriented Bounding Box (OBB) Detection, and Keypoint Detection, making it a highly versatile framework for detailed geometric analysis.

This core architecture serves as a blueprint for a family of models of varying sizes, ranging from nano (n) to extra-large (x). The scaling is controlled by adjusting the number (n) of blocks or the number of channels. This provides a spectrum of models that balance the trade-off between performance and computational cost. Smaller models, like *YOLOv11n-seg*, are lightweight (2.9 M parameters) and optimized for high-speed inference, making them ideal for resource-constrained environments. In contrast, larger models, such as *YOLOv11x-seg*, leverage a much greater number of parameters (62.1 M) to achieve state-of-the-art accuracy.

#### 2.2.2. Image Dataset and Domain Adaptation

The development of a robust damage identification model requires a comprehensive and diverse dataset that reflects real-world inspection scenarios. To achieve this, this study utilizes a multi-source dataset approach, leveraging both publicly available and custom-collected imagery to systematically train and fine-tune the YOLO11 architecture. The datasets were selected and curated to address the specific task of identifying three primary damage classes: cracks, efflorescences, and exposed rebars.

The foundational model weights were sourced from a network pre-trained on the Microsoft Common Objects in Context (MS COCO) dataset. This provides a powerful starting point, as the model has already learned a robust hierarchy of general visual features, which significantly accelerates convergence during task-specific fine-tuning.

For the initial stage of damage-specific training, a curated subset of the public DACL10K bridge damage dataset was employed, which is publicly available in [27]. From the original 9920 images in the collection, a dedicated set of 3737 images was selected. This curation process involved filtering the dataset to include only images containing the three target damage classes. This public dataset, sourced from over 100 distinct bridges, ensures that the model learns to identify a wide variety of damage morphologies under different conditions.

**Figure 7:**
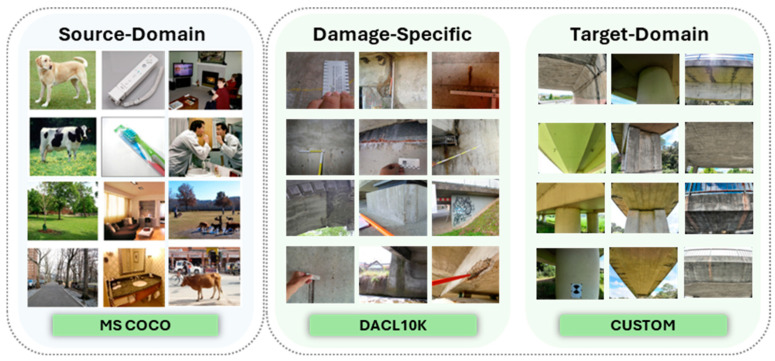
Samples of the Image Datasets.

**Figure 8:**
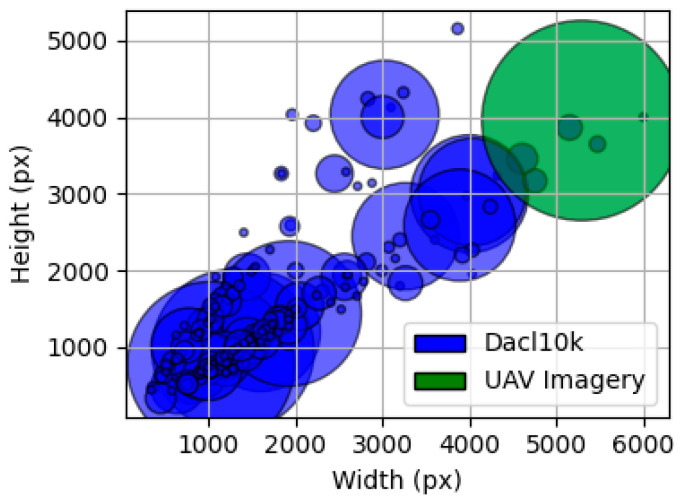
Shift resolution between the public DACL10 K dataset (blue) and the custom UAV imagery (green).

The final and most critical dataset is a private, custom-collected (20 MP) high-resolution UAV dataset (5280 × 3956 px). This collection consists of 645 images for training and 161 images for validation, all captured via Unmanned Aerial Vehicles (UAVs) during structural inspections. To ensure a rigorous and unbiased evaluation of all models and strategies, this 161-image validation set was strictly held out from all training and hyperparameter tuning processes. It therefore served as an independent test set for reporting all final performance metrics presented in this study. These high-resolution images were annotated for cracks, efflorescences, and exposed rebars using the Label Studio 1.19 software. This custom dataset represents the target domain for the model and is essential for the final fine-tuning stage, which optimizes the model’s performance for the unique high-resolution, top-down perspective, and variable lighting conditions characteristic of an aerial inspection workflow. Figure 7 illustrates samples from these three datasets, while Figure 8 demonstrates the shift resolution faced in the dataset.

#### 2.2.3. Training and Optimization Strategy

The training methodology was designed to systematically bridge the significant domain gap between the damage-specific public datasets and the target-domain high-resolution inspection imagery. This multi-stage approach was crucial for mitigating the risk of overfitting and enhancing generalization, given the limited size of the final target-domain dataset. The problem of domain shift, where a model’s performance degrades when the target data distribution differs from the training data, is a well-documented challenge [28,29] and is analogous to the broader issues of environmental and operational variability in the field of Structural Health Monitoring [30]. In this application, a primary cause of this shift is the mismatch in image resolution, which fundamentally alters the scale at which damage features are represented. To address this, a multi-stage transfer learning and domain adaptation strategy was developed. The implementation is based on the public Ultralytics framework [31] and custom scripts developed for this analysis.

**Figure 9:**
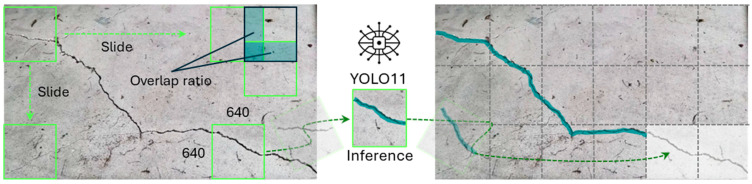
Slicing Aided Hyper Inference (SAHI) process for high-resolution imagery.

To establish a consistent and optimized protocol for the subsequent comparative analysis, a preliminary study was conducted to identify the most effective training approach. The performance of three distinct experimental configurations was systematically evaluated against the consistent validation set of 161 high-resolution images, which was held out from all training data to ensure an unbiased comparison. The first configuration established a baseline by fine-tuning the model solely on the curated subset of the public DACL10K dataset. The second, a direct Domain Adaptation approach, involved fine-tuning the model on the custom UAV imagery after resizing it to a standard input resolution. The third configuration utilized a Slicing Aided Hyper Inference (SAHI) approach (Figure 9). Essentially, this technique partitions each high-resolution image into a set of overlapping tiles that match the model’s native input size. For this study, we employed a tile size of 640 × 640 pixels with a 20% overlap ratio between adjacent tiles, thereby preserving the fine-grained visual features essential for detecting small-scale defects.

The results of this preliminary study, detailed in the Discussion and Results section, informed the selection of the optimal training strategy. The final, optimized configuration was then adopted as the standardized training protocol for all YOLO11 architectures evaluated in this work, ensuring a rigorous and equitable benchmark.

#### 2.2.4. Activation Map Generation

The critical link between the detection and segmentation stages of the proposed framework is the generation of a class-specific activation map. For each object instance identified by the YOLO11 model, a raw probability map is extracted directly from the model’s instance segmentation head. This approach is fundamentally different from and more informative than using the detector’s final binary mask or its bounding box as a prompt.

**Figure 10:**
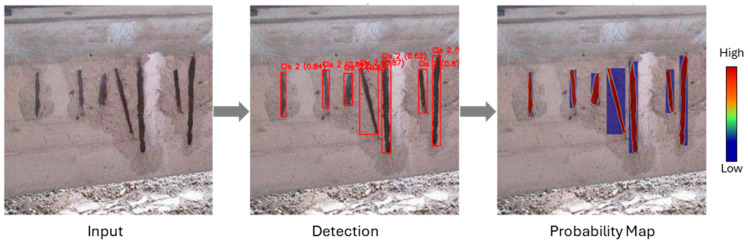
Probability map generation process.

Unlike a finalized binary mask, which contains only absolute values representing “object” or “background,” the raw map is a single-channel, where each pixel’s intensity (ranging from 0.0 to 1.0) corresponds to the model’s confidence that the pixel belongs to the detected damage instance. This probabilistic output is accessed by temporarily modifying the standard mask processing function within the YOLO11 framework. This technical step intercepts the model’s output before the final confidence thresholding is applied, thereby preserving the rich, granular data about the model’s certainty across the entire detected region. Figure 10 illustrates this process.

This activation map serves as a more effective guide for the subsequent segmentation stage than a simple bounding box for several reasons. The reason is that a bounding box provides only a coarse, rectangular approximation of the damage, which is particularly inefficient for geometrically complex or elongated defects like cracks. The activation map, by contrast, provides a detailed spatial distribution of the model’s confidence, naturally conforming to the shape of the damage. The high-activation regions directly correspond to the visual features that the model has learned are most discriminative for identifying a specific damage class, making it a highly reliable and contextually aware source for generating precise prompts.

### 2.3. Stage 2: Point-Guided SAM Segmentation

#### 2.3.1. Segment Anything Model (SAM) Architecture

**Figure 11:**
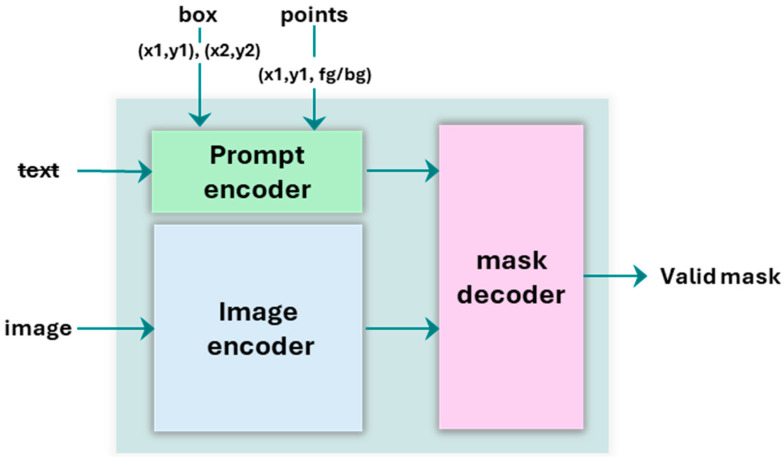
SAM model architecture.

The Segment Anything Model (SAM) [24] is a foundational model for image segmentation, engineered to perform zero-shot generalization for a vast range of objects and image types without the need for task-specific fine-tuning. Pre-trained on an extensive dataset of over one billion masks, SAM is designed as a promptable system that generates high-quality segmentation masks from simple user-provided cues, such as points or bounding boxes, as depicted in Figure 11. This capability allows it to address the critical data bottleneck often encountered in supervised deep learning by enabling data-efficient, high-fidelity segmentation. The architecture is structured around three key components that work together: an Image Encoder, a Prompt Encoder, and a Mask Decoder.

**Image Encoder.** The core of SAM’s powerful representational ability lies in its Image Encoder, which is built upon a Vision Transformer (ViT) architecture. This heavyweight component processes a high-resolution input image and generates a comprehensive, high-dimensional embedding. This embedding encapsulates the complex spatial relationships and semantic features of the entire image, creating a rich internal representation that allows the model to understand the content robustly before any specific segmentation task is requested.

**Prompt Encoder.** The Prompt Encoder is a lightweight module responsible for efficiently converting various forms of user guidance into vector embeddings. It is designed to handle sparse prompts, such as text, point coordinates and bounding boxes. For this study, it specifically utilizes positional encodings to represent the spatial locations of foreground (positive) points, background (negative) points, and bounding boxes. This encoding process translates these discrete spatial cues into a high-dimensional representation that is readily interpretable by the subsequent Mask Decoder.

**Mask Decoder.** The Mask Decoder is a fast and lightweight transformer-based network that integrates the information from both the image and prompt embeddings to predict the final segmentation mask. It is designed to be highly efficient, allowing for real-time mask generation as prompts are provided. The decoder is also ambiguity-aware; if a prompt could plausibly refer to multiple nested objects, it has the capacity to output multiple valid masks. However, the precise nature of the multi-point prompting strategy employed in this work is designed to minimize such ambiguity and guide the model toward a single, accurate output.

#### 2.3.2. Prompting Strategies for SAM

The efficacy of the Segment Anything Model is fundamentally dependent on the quality and nature of the prompts used to guide its segmentation process. The model’s flexible design accommodates various forms of user guidance, including bounding boxes, text, and discrete points. This study systematically evaluates different prompting strategies to determine the most effective method for leveraging the output of the foundational YOLO11 detector to achieve high-fidelity damage segmentation.

The most conventional approach is the use of a bounding box prompt. In this strategy, the rectangular coordinates generated by the YOLO11 detector are directly fed to SAM as a spatial prior. While straightforward, this method is often suboptimal for structural damages. For geometrically complex or elongated defects such as cracks, a bounding box is an inefficient prompt as it contains a large percentage of background pixels, providing a noisy and ambiguous signal that can degrade SAM’s performance.

An alternative is the use of text prompts, where a class label like “crack” is used as input. However, this approach lacks the precise spatial information required to distinguish between multiple instances of the same damage class within a single image, making it unsuitable for a comprehensive inspection workflow.

To overcome these limitations, this study evaluates an optimized point-prompting strategy. This method is designed to translate the rich, probabilistic information from the YOLO11 activation map into a set of precise and informative spatial cues. The algorithm, summarized in Algorithm 1, is executed for each detected damage instance and is designed to be robust against variations in the size and shape of the defects. The procedure begins by isolating the activation map for each detected instance using its corresponding bounding box, which ensures the analysis is focused solely on the region of interest. To create a selection process that is invariant to the original scale and aspect ratio of the damage, the cropped activation map is then resized to a standard, fixed-size grid (e.g., 100 × 100 pixels). This size standardization is a critical step, as it allows the point selection and filtering logic to operate on a consistent and normalized spatial domain.
**Algorithm 1:** Optimized Point-Prompt Generation**Input:** A raw probability map and its corresponding bounding box.**Output:** A set of point coordinates and labels (fg/bg) for SAM prompting.**1. Crop Map:**Crop the probability map using the bounding box coordinates.**2. Standardize Size:**Resize the cropped map to a standard grid (e.g., 100 × 100 pixels).**3. Identify Candidate Points:**Identify candidate points by thresholding the standardized grid (e.g., >0.9 for positive, <0.7 for negative).**4. Filter Points:**Filter candidate points to select a sparse subset based on confidence and Euclidean distance(e.g., 20 pixels).**5. Transform Coordinates:**Transform filtered point coordinates from the standard grid back to the original image space.**6. Final Output:**Combine final points and generate corresponding labels (1 for foreground, 0 for background).

**Figure 12:**
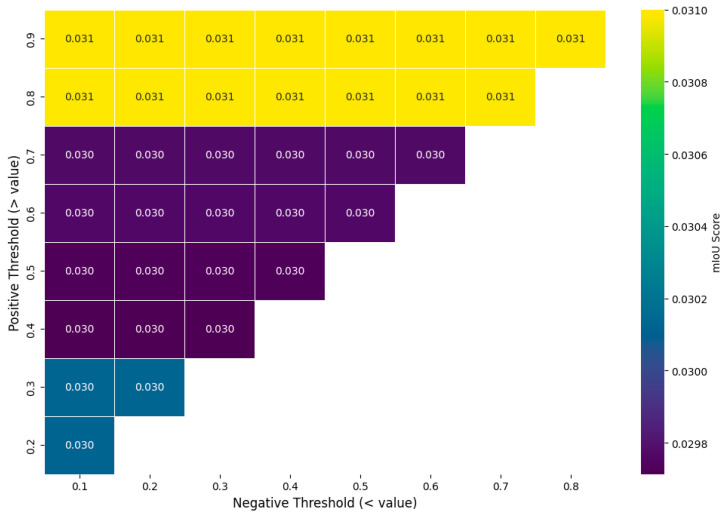
Sensitivity analysis of point-prompting thresholds. The heatmap shows the mIoU score for different combinations of positive (y-axis) and negative (x-axis) thresholds.

Within this standardized grid, candidate prompt points are identified by applying confidence thresholds. To empirically determine the optimal values and avoid arbitrary selection, a sensitivity analysis was conducted. As illustrated in Figure 12, the mean Intersection over Union (mIoU) score was evaluated across a range of positive (foreground) and negative (background) threshold pairs. The analysis reveals that segmentation performance is highly sensitive to the positive threshold, with the highest mIoU score (0.031) consistently achieved when selecting only the most confident pixels (positive threshold > 0.8). Based on this empirical evidence, a positive threshold of 0.9 and a negative threshold of 0.7 were selected for the point-prompting experiments to balance high performance with sufficient background context, thus validating the methodological choice.

These candidate points are sorted by their confidence scores and then subjected to a distance-based filtering algorithm (e.g., 20 pixels). This process selects a sparse, well-distributed set of the most confident points, preventing the submission of a dense, uninformative cluster of prompts. Finally, the coordinates of these refined prompt points undergo a coordinate transformation, scaling them from the standardized grid space back to their correct locations on the original, full-resolution input image. This final set of foreground and background points constitutes the precise prompt that is fed to SAM to generate the high-fidelity segmentation mask. Algorithm 1 summarizes the algorithm for optimized point-prompt generation.

### 2.4. Evaluation Metrics

The performance of the YOLO11 model architectures and the proposed framework is evaluated using a standard and robust set of metrics for instance segmentation. The evaluation is grounded in the concepts of True Positives (TP), a correct detection of an actual damage instance; False Positives (FP), an incorrect detection where no damage exists; and False Negatives (FN), an actual damage instance that the model failed to detect.

The classification of a prediction as a True Positive is determined by the Intersection over Union (IoU). The IoU measures the spatial overlap between the predicted area ($A_{pred}$) of the bounding box (b) or mask (m) and the area of the ground-truth annotation ($A_{gt}$), ensuring that a detection is not only correctly classified but also accurately localized. A prediction is typically considered a TP if its IoU with a ground-truth mask exceeds a predefined threshold. The IoU is calculated as

(1) $$\mathrm{IoU}=\frac{\mathrm{Area}\left(A_{pred}\cap A_{gt}\right)}{\mathrm{Area}\left(A_{pred}\cup A_{gt}\right)}$$

Based on the counts of *TP*, *FP*, and *FN*, three fundamental metrics are computed. Precision quantifies the accuracy of predictions, reflecting the proportion of correct detections among all predictions, while Recall measures the ability of the model to detect all actual instances of damage. The *F1*-score represents the harmonic mean of Precision (*P*) and Recall (*R*), providing a balanced measure of performance. These metrics are defined as

(2) $$P=\frac{TP}{TP+FP}$$

(3) $$R=\frac{TP}{TP+FN}$$

(4) $$F1=2\times \frac{P\times R}{P+R}$$

The primary benchmark for this study is the mean Average Precision (mAP), which provides a single, comprehensive score summarizing the model’s performance across all classes and confidence levels. The mAP is computed as the mean of the class-specific Average Precision (AP) scores. The AP for each class is formally defined as the area under the Precision-Recall curve, calculated by integrating precision over the change in recall:

(5) $$\mathrm{AP}=\int_{0}^{1}P\left(R\right)\;dR$$

To provide a thorough evaluation, two standard mAP variants are reported: mAP50, which is the mAP calculated at a single IoU threshold of 0.50, and mAP50-95, which offers a more stringent evaluation by averaging the mAP score over ten different IoU thresholds from 0.50 to 0.95.

## 3. Discussion and Results

This section presents the results as a progressive ablation study to isolate the contribution of each methodological component. Section 3.1 first identifies the optimal base architecture. Section 3.2 then establishes a performance baseline and quantifies the impact of domain adaptation. Subsequently, Section 3.3 evaluates the effect of a high-resolution processing strategy (SAHI), leading to the final hybrid pipeline, which is optimized and analyzed in Section 3.4.

### 3.1. Comparative Analysis of YOLO11-Seg Variants Models

To establish the most effective foundation for the initial segmentation stage, a comparative analysis was conducted across the scaled architectures of the YOLO11-seg series. The YOLO11 family offers a spectrum of models with varying sizes, specifically Nano (n), Small (s), Medium (m), Large (l), and Extra-Large (x), which present a fundamental trade-off between model complexity and performance. Larger models, such as YOLOv11x-seg, contain more parameters, allowing them to learn more intricate features and achieve higher segmentation accuracy. However, this increased complexity comes at the cost of greater computational requirements, leading to longer training times. Conversely, smaller models like YOLOv11n-seg are lightweight and computationally less expensive but may yield lower accuracy.

For this analysis, five pre-trained models (in MS COCO) were selected for fine-tuning: yolo11n-seg.pt, yolo11s-seg.pt, yolo11m-seg.pt, yolo11l-seg.pt, and yolo11x-seg.pt. Each model was fine-tuned for 50 epochs on the subset of the public DACL10K bridge damage dataset. To ensure a fair and direct comparison, the training was conducted on an NVIDIA RTX 4090 GPU with a batch size of 4 images. Across all experiments, the model was trained using the Adam optimiser with an initial learning rate of 10-2, which was linearly reduced to 10–4 over the training duration, and a momentum factor of 0.9. To enhance the model’s robustness and generalisation capabilities, a suite of data augmentation techniques was applied, including mosaic augmentation, random horizontal flips (with a 0.5 probability), random scaling (±0.5), copy and paste flip instance and colour space adjustments to hue (±0.015), saturation (±0.7), and value (±0.4).

**Figure 13:**
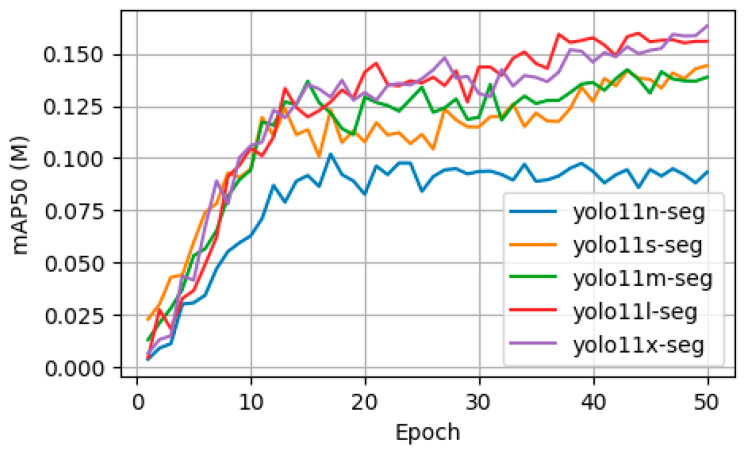
Training Convergence of YOLO11-seg Variants.

The results of the training reveal a clear relationship between model size, segmentation accuracy, and computational cost. The largest model, YOLO11x-seg, achieved the highest final segmentation accuracy with a mAP50 of 0.16319. The training graph in Figure 13 shows that larger models, particularly the large (l) and extra-large (x) variants, reached higher peak performance. However, this superior accuracy comes at a significant computational cost. As shown in Table 1, the training time increased with model size, with YOLO11x-seg requiring 99.19 min, compared to just 66.03 min for the smallest YOLO11n-seg model.

Given that the primary objective of this framework is to generate the highest-fidelity probability maps to serve as precise prompts for the Segment Anything Model, achieving maximum initial segmentation accuracy was prioritized over minimizing training time. The higher and more stable performance of the larger models ensures the most reliable input for the second stage of the framework. Therefore, the YOLO11x-seg model was selected as the baseline architecture for all subsequent experiments in this study. This trend, clearly correlated with the model parameter counts presented in Table 1, suggests that for the specific challenge of segmenting subtle and varied damage patterns in high-resolution aerial imagery, a deep architecture with a high parameter count is critical for achieving state-of-the-art accuracy, and that a shallower model would likely be insufficient.

### 3.2. Analysis of Domain Adaptation and End-to-End Segmentation Strategies

Having established the YOLOv11x-seg as the most accurate foundational architecture, the subsequent analysis evaluates the most effective end-to-end strategy for generating high-fidelity instance segmentation masks. For this stage of the investigation, all models were trained and evaluated on images resized to a fixed 640 × 640 pixel resolution to establish a performance benchmark under conventional processing constraints. This evaluation follows a progressive methodology, beginning with an assessment of the foundational model’s performance before (only DACL10K, called baseline) and after fine-tuning on the custom UAV dataset to quantify the impact of domain adaptation. Following this, the output of the optimized, domain-adapted model is used to conduct a comparative test of three distinct segmentation strategies: the model’s own native segmentation output, the conventional SAM framework prompted by bounding boxes, and the proposed framework using SAM prompted by points derived from the probability map.

All strategies were benchmarked on the 161-image high-resolution validation set, which was resized accordingly, with the results presented in Table 2. The initial baseline, representing the YOLOv11x-seg model trained only on the public DACL10K dataset, performed poorly when evaluated on the target-domain UAV imagery, achieving an mIoU of just 0.014. This result underscores the significant domain shift between the datasets and confirms that models trained on general public data are not directly applicable to specialized, high-resolution inspection tasks, especially after significant down-scaling. After fine-tuning on the 645 custom UAV images, a dramatic improvement in performance was observed. The domain-adapted model’s native segmentation output achieved an mIoU of 0.118, an over eightfold increase from the baseline. This demonstrates that adapting the model to the specific visual characteristics of the target dataset is the single most critical factor in achieving a functional foundational model, even when image resolution is compromised.

With this robust, domain-adapted model as the new baseline, the two SAM-based refinement strategies were evaluated. The results, however, reveal a critical limitation of applying these advanced segmentation frameworks to severely down-scaled imagery. Both the bounding box and point-prompted SAM strategies underperformed relative to the native output of the domain-adapted YOLO11 model. The SAM with bounding box prompts achieved an mIoU of 0.098, while the proposed point-prompting strategy yielded an mIoU of only 0.031. This counterintuitive decrease in performance suggests that the substantial loss of detail from resizing the high-resolution UAV images to a 640 × 640 resolution renders the prompts ineffective. The probability maps generated from the low-resolution images are likely too diffuse to provide reliable points, and the object boundaries become too indistinct for a bounding box to serve as a precise guide for SAM. These findings indicate that while domain adaptation is essential, the potential of sophisticated two-stage frameworks like the one proposed is fundamentally constrained by the quality of the input resolution. This motivates the investigation in the following section, which explores a training and inference strategy that preserves the native high resolution of the imagery.

### 3.3. Performance Enhancement with Slicing Aided Hyper Inference (SAHI)

The preceding analysis established that severely down-scaling high-resolution UAV imagery to a fixed 640 × 640 input resolution acts as a critical performance bottleneck. This process discards essential high-frequency details, fundamentally limiting the efficacy of the foundational detector and rendering advanced prompting strategies for SAM ineffective. To overcome this limitation, this final stage of the investigation evaluates the impact of a training and inference strategy that preserves the native resolution of the imagery: Slicing Aided Hyper Inference (SAHI). With this approach, high-resolution images are partitioned into overlapping tiles (20%) that match the model’s native input size, ensuring that no critical visual information is lost to down-scaling. The three end-to-end segmentation strategies, native model output, SAM with bounding box prompts, and SAM with point prompts, were re-evaluated using the foundational YOLOv11x-seg model, which was fine-tuned using the full SAHI protocol.

The results, presented in Table 3, demonstrate a dramatic and substantial performance enhancement directly attributable to the SAHI methodology. By training on high-resolution tiles, the foundational detector learned more discriminative and fine-grained features, causing the performance of the crack class to increase compared to the resized-image experiments. More importantly, this high-fidelity context revealed a more nuanced, class-dependent behaviour among the different segmentation strategies that was previously obscured by the low-resolution data.

The quantitative results highlight that no single strategy is optimal across all damage types. For geometrically complex and elongated defects like cracks, the native, domain-adapted SAHI model demonstrates superior performance (IoU of 0.213) compared to both SAM-based approaches. This is because a bounding box provides an inefficient and ambiguous prompt for such features. A tight bounding box around a thin crack contains a large percentage of background pixels, which provides a noisy signal to SAM’s generalist segmentation engine and can result in incomplete or inaccurate masks. Conversely, the native YOLO11 segmentation head is trained end-to-end specifically on this pathology, allowing it to develop a more effective internal representation for delineating these fine, linear features directly.

Conversely, for damage types that are better-contained with more defined boundaries, such as efflorescence and exposed rebar, the two-stage framework using SAM with a Bounding Box Prompt shows a clear advantage, achieving the highest IoU scores for both classes, IoU of 0.140 and 0.092, respectively. This indicates that for less intricate shapes, the bounding box provides a sufficient spatial prior for SAM’s segmentation engine to effectively refine the boundaries of the detected region. The proposed point-prompting strategy, while conceptually sound, did not yield competitive results, suggesting that the generated probability maps, even at high resolution, may not be sufficiently sharp or that the point-selection algorithm requires further refinement.

Based on this comprehensive analysis, the optimal strategy is not a monolithic approach but a hybrid, class-specific pipeline that leverages the respective strengths of the specialist detector and the foundational model.

### 3.4. Final Strategy and Qualitative Analysis

It is important to address the exclusion of the point-prompting strategy from this final pipeline, despite its initial focus in the methodology. The initial hypothesis was that precise point prompts derived from the model’s activation map would outperform bounding boxes. However, the experimental results consistently demonstrated the opposite. As detailed in Section 3.2 and Section 3.3, the point-prompting strategy yielded the lowest performance under both resized and high-resolution SAHI conditions. This suggests that the generated probability maps, while useful internally for the model, may not be sufficiently sharp for reliable external prompting, or that the point-selection algorithm itself requires more advanced refinement. Therefore, based on this clear empirical evidence, the final hybrid model was constructed using only the strategies that proved most effective in practice: the native detector output and the bounding box-prompted SAM.

**Figure 14:**
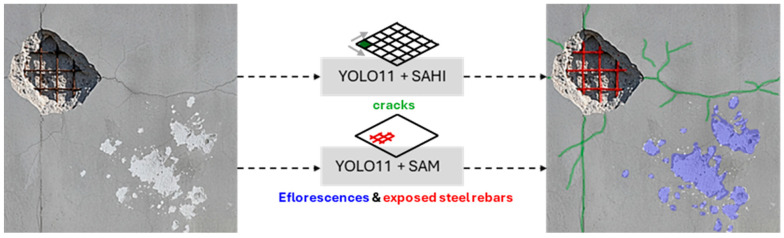
Illustration of the proposed hybrid segmentation strategy.

The findings from the comparative analysis culminate in a final, optimized hybrid strategy illustrated in Figure 14. The proposed pipeline dynamically assigns the segmentation process according to the identified damage class. Specifically, for cracks, the segmentation results produced directly by the domain-adapted, SAHI-trained YOLOv11x-seg model are employed. In contrast, for Efflorescence and Exposed Rebar, the bounding box predictions generated by the same model are used as prompts for the Segment Anything Model (SAM), which subsequently produces the final segmentation output. In practice, this hybrid routing is implemented as conditional logic in the post-processing script. The YOLOv11x-seg model first generates predictions, each containing a class label, a native segmentation mask, and a bounding box. The script then iterates through each detection; if the predicted class label is ‘crack’, the native mask is retained. If the label is ‘efflorescence’ or ‘exposed rebar’, the script discards the native mask and uses the detection’s bounding box to prompt SAM, using the resulting SAM-generated mask as the final output for that instance.

To maximize the efficacy of this hybrid approach, a final hyperparameter optimization was performed using the Ray Tune library. This process revealed several key adjustments to the training configuration. It identified Stochastic Gradient Descent (SGD) as the optimal optimizer for the final fine-tuning stage. Furthermore, the optimization indicated that several common data augmentation techniques, including mosaic, random scaling, translation, and horizontal flips, were counterproductive. Disabling them for the final fine-tuning on the highly specific UAV dataset prevented the distortion of fine-grained features, leading to a more robust model. The final optimized training configuration that produced these results is detailed in Table 4.

The implementation of this optimized hybrid strategy resulted in a substantial boost in performance. As detailed in Table 5, the model achieved a final mAP50 (box) of 0.602 and a more stringent mAP50-95 (box) of 0.361 on the same validation set, whereas mAP50 (mask) of 0.582 and mAP50-95 (mask) of 0.281, resulting in an average mAP50 of 0.593. The final segmentation IoU for cracks reached 0.495, while the IoUs for efflorescence and exposed rebar were 0.331 and 0.205, respectively.

**Figure 15:**
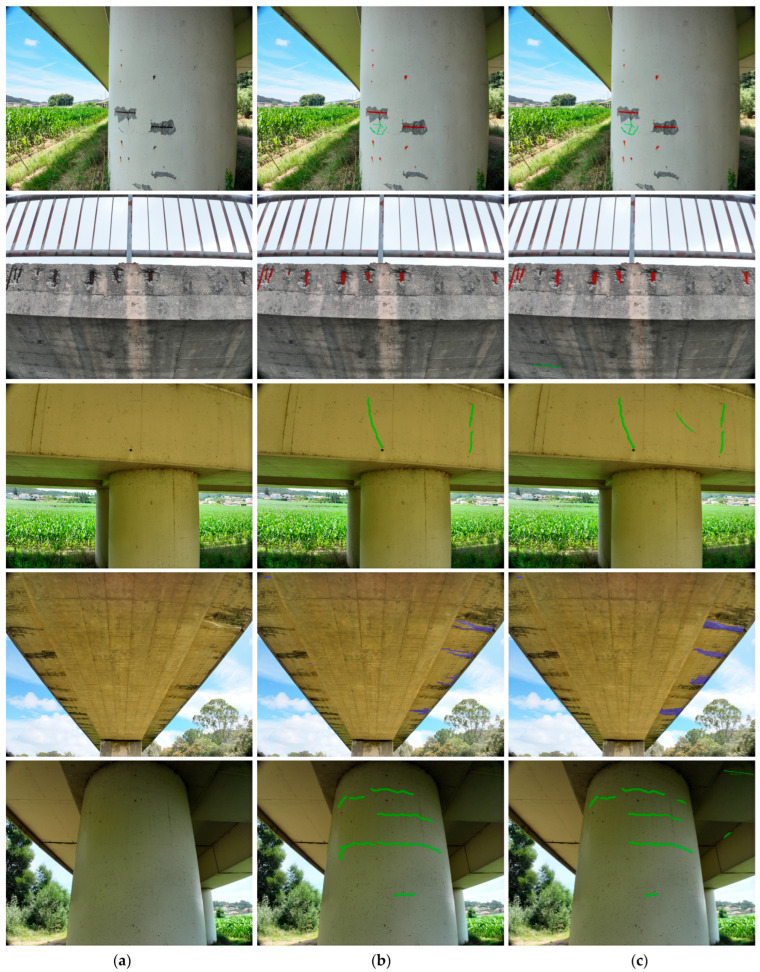
Qualitative results of the final hybrid model on sample images (cracks shown in green, exposed steel rebars in red, efflorescences in blue): (**a**) Original Image, (**b**) Ground Truth Annotation, (**c**) Model Prediction.

A qualitative analysis provides further insight into the practical performance of the proposed hybrid pipeline. Figure 15 presents a selection of results from the validation set, comparing the model’s predictions to the ground truth annotations. In the first example (top row), the model accurately delineates a complex network of fine cracks and correctly segments a large area of exposed rebar, demonstrating the successful application of both branches of the hybrid strategy. The following examples demonstrate the model’s ability to handle challenging lighting conditions and diverse damage morphologies, and in some cases, even to identify damage missed by human ground truth.

However, the analysis also reveals limitations. In some cases, very fine hairline cracks are missed, and the boundaries of efflorescence can be ambiguous where the damage fades gradually into the concrete substrate, which helps explain the comparatively lower IoU scores for this damage class. A similar challenge occurs with exposed rebar, where defining the precise boundary between the bars and the surrounding irregular spalling leads to lower IoU scores. These instances highlight areas for future improvement, such as the potential integration of more advanced prompt-generation techniques or further refinement of the model architecture. Nonetheless, the qualitative results confirm that the proposed hybrid strategy provides a robust and effective solution for segmenting multiple classes of structural damage in high-resolution aerial imagery.

## 4. Conclusions

This study introduced and validated a novel hybrid deep learning pipeline that synergizes a YOLOv11x-seg detector with the Segment Anything Model (SAM) for multi-damage segmentation of cracks, efflorescences, and exposed rebars in high-resolution UAV inspection imagery. The findings demonstrate that a class-specific approach is optimal, as no single segmentation strategy proved globally effective for all damage types. Specifically, it was established that the final hybrid model achieved a robust mean Average Precision (mAP50) of 0.593, with the native YOLO output yielding a superior Intersection over Union (IoU) of 0.495 for linear cracks, while a SAM-based approach was more effective for efflorescence (0.331 IoU) and exposed rebar (0.205 IoU). Furthermore, the analysis revealed the critical importance of Slicing Aided Hyper Inference (SAHI) in overcoming the performance bottleneck caused by down-scaling high-resolution images, which provides new insights into processing aerial inspection data.

The primary implication of this work is the potential for more accurate and reliable automated structural health monitoring systems. By providing a more robust and context-aware solution, this research paves the way for intelligent inspection frameworks that dynamically leverage the respective strengths of specialized detectors and powerful foundation models. These findings will be valuable for researchers and practitioners in the field of automated structural inspection and computer vision in civil engineering.

However, the study acknowledges several limitations that offer avenues for future research. The current study was conducted using a custom high-resolution dataset focused primarily on concrete bridge infrastructure. Therefore, the generalizability of the findings to different types of structures or materials under varied environmental conditions needs further investigation. Furthermore, while this study provides a deep comparison of strategies within the YOLO/SAM framework, it does not include benchmarks against other established segmentation architectures like Mask R-CNN or DeepLabv3+. Future work should include such a comparison to more broadly validate the superiority of the proposed hybrid approach. Additionally, the proposed point-prompting strategy for SAM, a key initial component of the investigation, did not outperform bounding box prompts, indicating that the prompt-generation algorithm requires further refinement to effectively translate the detector’s confidence maps into reliable spatial cues for SAM. Finally, the proposed hybrid pipeline, while effective, introduces computational complexity not fully explored in this study. The use of SAHI is resource-intensive, and the class-conditional branching adds a layer of logic to the inference process.

Future work will focus on addressing these limitations. The immediate goal is to investigate more advanced prompt-generation techniques to enhance the performance of point-guided segmentation for all damage classes. Subsequently, it is planned to explore the integration of this hybrid pipeline into a real-time, on-board processing system for UAVs to provide immediate field-level analysis. These efforts will further enhance the robustness and utility of the developed technology, contributing to the advancement of intelligent infrastructure monitoring.

## Figures and Tables

**Table 1:** Comparative Performance and Training Time of YOLOv11-seg Variants.

Model	Params (M)	mAP50 @ Epoch 50	Time (min)
yolo11n-seg	2.9	0.09329	66.03
yolo11s-seg	10.1	0.14422	65.88
yolo11m-seg	22.4	0.13872	81.13
yolo11l-seg	27.6	0.15593	98.68
yolo11x-seg	62.1	**0.16319**	99.19

**Table 2:** Comparative Performance of End-to-End Segmentation Strategies.

Strategy	Input Resolution	IoU_crack_	IoU_Efflor_	IoU_Exp.Rebar_	mIoU
Baseline	Resized (640 × 640)	0.003	0.007	0.031	0.014
Domain-Adapted	Resized (640 × 640)	**0.165**	0.123	0.065	0.118
SAM with BBox Prompt	Resized (640 × 640)	0.061	**0.140**	**0.092**	0.098
SAM with Point Prompts	Resized (640 × 640)	0.014	0.053	0.026	0.031

**Table 3:** Final Performance Enhancement with SAHI-based Training.

Strategy	Input Resolution	IoU_crack_	IoU_Efflor_	IoU_Exp.Rebar_	mIoU
Domain-Adapted	Native High-Resolution	**0.213**	**0.059**	**0.070**	0.114
SAM with BBox Prompt	Native High-Resolution	0.095	0.043	0.065	0.068
SAM with Point Prompts	Native High-Resolution	0.025	0.031	0.055	0.037

**Table 4:** Hyperparameter Settings for the Final Optimized Model.

Hyperparameter	Value
Optimizer	SGD
Initial Learning Rate	0.01
Final Learning Rate Factor	0.001
Momentum	0.937
Weight Decay	0.0005
Epochs	100
Batch Size	4
Input Tile Size (SAHI)	640 × 640 pixels
Augmentations	Disabled

**Table 5:** Final Performance of the Optimized Hybrid Strategy.

Strategy	Input Resolution	IoU_crack_	IoU_Efflor_	IoU_Exp.Rebar_	mAP50	mAP50-95
Domain-Adapted	Native	0.495	-	-	0.585 (m)	0.281 (m)
SAM w/BBox Prompts	Resized (640 × 640)	-	0.331	0.205	0.602 (b)	0.361 (b)

## Data Availability

Code and Datasets may be available upon request.
